# Challenges and Recent Advances in Enzyme-Mediated Wastewater Remediation—A Review

**DOI:** 10.3390/nano11113124

**Published:** 2021-11-19

**Authors:** Khadega A. Al-Maqdi, Nada Elmerhi, Khawlah Athamneh, Muhammad Bilal, Ahmed Alzamly, Syed Salman Ashraf, Iltaf Shah

**Affiliations:** 1 Department of Chemistry, College of Science, United Arab Emirates University, Al Ain P.O. Box 15551, United Arab Emirates; 200935138@uaeu.ac.ae (K.A.A.-M.); ahmed.alzamly@uaeu.ac.ae (A.A.); 2 Department of Biology, College of Arts and Sciences, Khalifa University, Abu Dhabi P.O. Box 127788, United Arab Emirates; 100052648@ku.ac.ae (N.E.); khawlah.athamneh@ku.ac.ae (K.A.); 3 Huaiyin Institute of Technology, School of Life Science and Food Engineering, Huaian 223003, China; bilaluaf@hotmail.com; 4 Center for Biotechnology (BTC), Khalifa University of Science and Technology, Abu Dhabi P.O. Box 127788, United Arab Emirates

**Keywords:** peroxidases enzymes, water remediation enzyme evolution, enzyme immobilization, hybrid nanoflowers, metal organic framework

## Abstract

Different classes of artificial pollutants, collectively called emerging pollutants, are detected in various water bodies, including lakes, rivers, and seas. Multiple studies have shown the devastating effects these emerging pollutants can have on human and aquatic life. The main reason for these emerging pollutants in the aquatic environment is their incomplete removal in the existing wastewater treatment plants (WWTPs). Several additional treatments that could potentially supplement existing WWTPs to eliminate these pollutants include a range of physicochemical and biological methods. The use of enzymes, specifically, oxidoreductases, are increasingly being studied for their ability to degrade different classes of organic compounds. These enzymes have been immobilized on different supports to promote their adoption as a cost-effective and recyclable remediation approach. Unfortunately, some of these techniques have shown a negative effect on the enzyme, including denaturation and loss of catalytic activity. This review focuses on the major challenges facing researchers working on the immobilization of peroxidases and the recent progress that has been made in this area. It focuses on four major areas: (1) stability of enzymes upon immobilization, enzyme engineering, and evolution; (2) recyclability and reusability, including immobilization on membranes and solid supports; (3) cost associated with enzyme-based remediation; and (4) scaling-up and bioreactors.

## 1. Introduction

**Figure 1:**
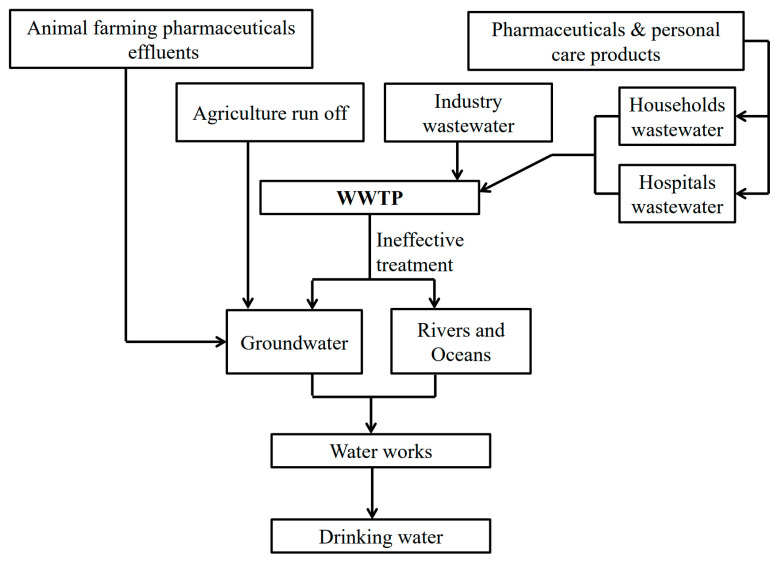
Potential pathways for emerging pollutants (Eps) to enter drinking water.

As the world population has continued to increase in recent times, a rapid rise in pollution has occurred around the world. New challenges and problems continue to affect the environment. Emerging pollutants (EPs) or contaminants of emerging concerns (CECs) are a serious problem [1,2,3]. Emerging pollutants include different classes of manmade organic compounds that are being detected in various water bodies around the world. These pollutants are not regulated by current water quality regulations. They include pesticides, pharmaceuticals (e.g., anti-inflammatory drugs (NSAIDs), antibiotics and analgesics), personal care products, hormones, and food additives [4,5]. Table 1 shows different classes of emerging pollutants and a short description of each. Many studies have shown that these pollutants can affect both aquatic and human life [4]. Some reports have shown increasing breast cancer risk and lower reproductive abilities in women [6]. For example, N,N-diethyl-meta-toluamide (DEET), which is used as an insect repellent, can lead to the inhibition of the central nervous system enzyme acetylcholinesterase in both mammals and insects [7]. Various studies have detected high concentrations of emerging pollutants in water bodies, such as lakes, rivers, and seas. Concentrations of emerging pollutants in different water bodies range from ng/L to a few hundred μg/L [8,9,10]. Pharmaceuticals have been found in different concentrations in ground water, including metoprolol (20–60 ng/L), ibuprofen (4.27–510 ng/L), bezafibrate (10–160 ng/L), and naproxen (3.19–2000 ng/L) [11]. Pesticides that have been found in Lake Vistonis include Alphamethrin, Fluometuron, Lambda-cyhalothrin and Lindane, with concentrations of 0.161 µg/L, 0.088 µg/L, 0.041 µg/L and 0.030 µg/L, respectively [12]. Not surprisingly, the presence of emerging pollutants in different water bodies around the world has attracted much attention from researchers and the scientific community. A literature review of some emerging pollutants found in high concentrations in water is summarized in Table 2. Emerging pollutants have devastating and damaging effects on the environment. Another issue with EPs is their continuous introduction to the ecosystem, which could offset their removal and transformation rate. The largest source of EPs in the ecosystem, including ground and drinking water, is the inadequate elimination of EPs from wastewater treatment plants (WWTPs). This happens because WWTPs are not designed to eliminate these chemical compounds, and thus, a significant quantity of EPs and their metabolites avoid elimination in WWTPs and enter aquatic ecosystems [1]. Figure 1 summarizes how these compounds enter water bodies. The challenge with emerging pollutants is that ecotoxicological and risk assessment data are not available. Additionally, we lack analytical methods to detect trace levels of emerging pollutants; consequently it is difficult to foresee their outcome in aquatic ecosystems. These challenges are being overcome with more toxicological and analytical research on these compounds. For example, liquid chromatography with mass spectrometry (LC–MS) and gas chromatography with mass spectrometry (GC–MS) are sensitive techniques for detecting EPs at trace concentrations in environmental matrices [4].

## 2. Removal of Emerging Pollutants by Various Methods

Many methods and approaches have been developed to eliminate emerging pollutants from different water bodies, such as drinking water, groundwater, and wastewater, and thus reduce their risk to human and marine life. The main approaches for the treatment of these pollutants are physical, chemical, and biological. In addition, there is a hybrid system in which two or more treatment approaches are used to eliminate emerging pollutants. Physical methods include filtration, coagulation, and adsorption. Chemical methods include advanced oxidation processes (AOPs), electrolysis, and ozonation. Biological methods involve the use of enzymes and bacteria [14,19].

### 2.1. Physiochemical Methods

Among the chemical methods, advanced oxidation processes (AOPs) are the most famous. This method uses an oxidizing agent that is activated during the reaction using ultraviolet (UV) light and ultrasound. Hydrogen peroxide (H_2_O_2_) is the main oxidizing agent used in AOPs. Treatments start with the formation of hydrogen peroxide radicals that initiate oxidation reactions against emerging pollutants (EPs), leading to the degradation of pollutants to less harmful and safer compounds [20,21]. A study showed that the degradation of sulfamethoxazole, an antibiotic, using UV light and H_2_O_2_, produced two intermediates during the reaction that were significantly less toxic to *L. stavia* seeds than sulfamethoxazole [22]. The disadvantages associated with the use of chemical methods include the production of a large amount of sludge, possible generation of harmful byproducts, and high operational costs [23]. For example, the use of ozone (O_3_) in the chemical method of ozonation can be costly due to the need for a continuous supply of O_3_, which has a short half-life of approximately 20 min [24].

Adsorption is a physical method used to remove emerging pollutants from water. Several types of sorbents can be used, such as activated carbon, wood chips, peat, zeolites, and silica gel. Adsorption techniques have been shown to be highly efficient in the elimination and removal of organic pollutants [25,26,27]. One advantage that gives adsorption a lead against other techniques is its low operational cost, which makes adsorption economically reasonable for industrial-scale-up. The most commonly used sorbent for organic pollutant removal from water is activated carbon. The effectiveness of this method depends on the type of carbon used and its amount [20,28].

Another physical method used for the removal of pollutants from water is membrane separation. This method has many advantages, including limited maintenance demands, simple operation, and easy installation. On the other hand, this method has some disadvantages, including high capital cost and the possibility of membrane clogging [29]. Today, membrane separation can be used for water treatment in textile plants if the water being treated has a small amount of dye [21]. Ion-exchange is another type of physical method. Ion exchange has some advantages, such as the ability to recover the solvent and the adsorbent. Its disadvantages are its high cost and low effectiveness [20].

Although physical and chemical methods are currently enjoying large-scale operation, they still have some challenges and limitations. The biggest challenges that these methods face are their high energy and operational costs, as well as a large amount of sludge being produced during the reactions.

### 2.2. Biological Methods

A greener alternative is the use of biological methods, which involve the utilization of enzymes and microorganisms, such as bacteria. Bioremediation is considered safer, environmentally friendly, less disruptive, and has a lower cost than physiochemical methods [30,31]. However, the use of biological methods for water remediation has some downsides. Harsh environmental conditions may disturb the organisms used and thus reduce the efficiency of biological methods [32].

#### 2.2.1. Microbial Bioremediation

The efficiency of microbial bioremediation depends on the activity and flexibility of the microorganisms. Multiple studies have demonstrated the ability of microorganisms to degrade emerging pollutants. Such studies have been carried out on different groups of bacteria to study their roles in the degradation of dyes, including azo dyes. The advantages of using bacteria in the degradation of dyes include low cost, ecofriendliness, and a reduced amount of sludge and mineralization [33,34]. For example, *Bacillus cereus* isolated from petroleum sludge was found to be capable of degrading different aromatic dyes such as reactive black 5, toluidine blue, ponceau BS, and Congo red. In the same study, *Bacillus cereus* was also shown to have the ability to degrade various emerging pollutants such as Fluometuron, Sulfamethoxazole, and Prometryn [35].

Generally, the degradation of azo dyes by bacteria requires the cleavage of the azo linkage using enzymes, such as laccase and azoreductase, that are produced during the beginning of the stationary phase in microbial growth [28]. Khan and Malik [36] were able to degrade 93% of reactive black 5 after incubation for 120 h with an isolated strain of *Pseudomonas entomophila* BS1. Another study investigated the degradation of four different azo dyes (reactive black 5, reactive orange 16, direct red 81 and disperse red 78) by *Pseudomonas rettgeri strain HSL1 and* *Pseudomonas species SUK1* [37]. Nnenna et al. [38] showed the ability of bacteria to degrade two antibiotics, ciprofloxacin and erythromycin. Ciprofloxacin is effective against Gram-negative bacteria, whereas erythromycin is effective against Gram-positive bacteria. These antibiotics have been found in wastewater treatment plants. Some isolated bacteria, such as *Pseudomonas* sp., *Micrococcus* sp., *Shigella* sp. and *Bacillus* sp., were successful in the biodegradation of these antibiotics. It was shown that variation in pH did not affect the degradation of these antibiotics by the bacterial strains. Other factors that have been shown to have a notable impact on treatment include oxygen, moisture, and lack of alternative sources of nitrogen and carbon. Physiochemical operational factors that can influence the bacterial degradation of pollutants include oxygen, pH, and the concentration of the pollutant as well as its structure, redox mediator, electron donor, temperature, and supplementary sources of nitrogen and carbon [32]. For example, oxygen can affect microbial degradation, as it plays a major role in cell growth, where it impacts the physiological characteristics of the cell. For pollutant degradation, excess oxygen may block this process by behaving as an electron acceptor. For anaerobic bacteria, oxygen is considered deadly and could inhibit azoreductase enzymes directly [39]. The best pH for dye degradation by bacteria is a neutral pH, and the rate of degradation declines dramatically when pH values are strongly acidic or alkaline [40].

In addition to the physiochemical factors, there are other biological factors that can affect the bioremediation process. For example, some inherent characters of microbes affect their ability to degrade different substrates, this might be attributed to the plasmid-encoded genes, which encode specific enzymes and provide specificity for substrates. Another factor is bacterial chemotaxis which is considered as an advantage to enhance the ability of bacteria to degrade recalcitrant organic compounds. Additionally, to efficiently degrade various compounds and enhance bioremediation process in general, complex interactive networks of different microbial communities may be required [41].

Two common strategies have been widely tested and discussed to improve the effectiveness of microbial bioremediation. One way is through the addition of pre-grown microbial cultures to enhance the degradation of contaminants; this is referred to bioaugmentation. Another way, called biostimualtion, is by injecting nutrients and other supplementary components to the native microbial population to induce propagation and growth. These strategies can improve the bioremediation efficiency by speeding up the degradation process [42]. 

Even though bioremediation is an excellent technology used to clean-up contaminated environments, there are still some gaps that need to be considered, e.g., the growth of these microorganisms, the mode of their action, the difficulty in culturing them, and the regulatory mechanisms operating in them. Such issues were tackled by the recent advances in in-silico analyses and omics technologies (e.g., metagenomics, transcriptomics, proteomics, and metabolomics), which made feasible for researchers to collect biological data about microbial communities’ inhabitating contaminated environments, their physiological and cellular mechanisms, and the enzymes associated with bioremediation. Hence, a multidisciplinary approach is essential to understand the chemistry and decipher the pathways to enhance environmental monitoring and bioremediation efficacies [43,44]. 

#### 2.2.2. Enzymatic Bioremediation (The Use of Peroxidase and Laccase Enzymes)

Enzymatic bioremediation is a method for water remediation. The most common enzymes for enzymatic bioremediation are oxidoreductases. This class of enzymes includes laccase and peroxidase enzymes.

Laccase enzymes are multicopper enzymes that are widely distributed in nature and found in plants and fungi. Laccase enzymes can be used in multiple biotechnology applications due to the oxidation capability of laccase enzymes for a wide range of phenolic and nonphenolic compounds. Laccase enzymes have been used extensively in water remediation applications [45,46]. Asadgol et al. successfully degraded bisphenol A, an endocrine disruptor, using purified laccase enzyme from *Paraconiothyrium variabile (PvL)*. After 30 min of treatment, the enzyme degraded approximately 60% of the pollutant [47]. Hongyan et al. [48] were able to degrade bisphenol A using the *Trametes versicolor* laccase enzyme. Auriol et al. [49] demonstrated the oxidation of three natural estrogens, estrone (E1), 17β-estradiol (E2), and estriol (E3), as well as a synthetic estrogen, 17α-ethinylestradiol (EE2), using laccase in both synthetic water and municipal wastewater effluent. In synthetic water, the optimum pH for the removal of these hormones was 6.0, however, this work focused on the effect of the wastewater matrix on laccase-mediated treatment. At pH 7 and a temperature of 25 ± 1 °C, the matrix of the wastewater did not significantly affect the treatment. In addition, 20 U/mL laccase catalyzed the complete removal of these hormones in both synthetic water and municipal wastewater. Triclosan is an antimicrobial agent that has been found in different water surfaces and sediments. In the absence of a redox mediator, the laccase enzyme was capable of degrading 56.5% of this pollutant within 24 h. When tested with two different redox mediators, 1-hydroxybenzotriazole and syringaldehyde, the degradation of triclosan improved to approximately 90%, resulting in the generation of low-molecular-weight transformation products. The toxicity of those transformation products was significantly reduced or nontoxic compared to the original pollutant [50]. In addition, many studies have demonstrated the use of laccase enzymes for the degradation and removal of various classes of aromatic dyes [51,52].

The other oxidoreductase enzymes used for water remediation are peroxidases. These enzymes are highly distributed in nature, largely in plants and microbes. Some of the distinctive properties of peroxidase enzymes are their high thermal stability and high redox potential. These enzymes are classified into two main groups: heme peroxidases and nonheme peroxidases. Heme peroxidase enzymes can oxidize a broad range of pollutants in the presence of hydrogen peroxide (H_2_O_2_) or other peroxides. The mechanism of this reaction starts with a native peroxidase (Fe^3+^) reacting with hydrogen peroxide to produce ‘Compound I’, which is a cation radical form of peroxidase (Fe^4+.^). This radical form, Compound I, can react with a large number of pollutants to produce radicals and then convert to the second form of the enzyme called ‘Compound II’, (Fe^4+^). This form of the enzyme, Compound II, can further react with pollutants and then return to its native form (Fe^3+^). Peroxidase enzymes have been intensively studied for their application in water remediation [53]. A large body of literature shows the ability of peroxidases to degrade toxic emerging pollutants into safer intermediates [54,55,56,57]. Al-Maqdi et al. [58] compared the ability of chloroperoxidase (CPO) relative to the UV light + H_2_O_2_ method to degrade a thiazole pollutant. The results demonstrated that the CPO enzyme produced intermediates that were significantly less toxic than the intermediate produced with the UV light + H_2_O_2_ method. This result supports the use of enzymes in water remediation as a better and safer alternative. Examples of peroxidases that have been used in water remediation include CPO, lactoperoxidase (LPO), and lignin peroxidase (LiP) [53]. Nevertheless, the use of enzymes can have some drawbacks, such as enzyme reusability and enzyme stability throughout water remediation. Some of these challenges can be overcome by enzyme immobilization.

## 3. Enzyme Immobilization

**Figure 2:**
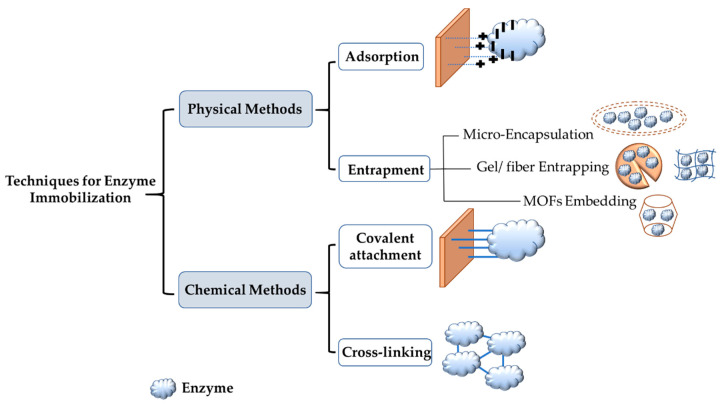
Commonly used approaches for enzyme immobilization.

Enzyme immobilization is defined as the attachment of an enzyme to an inert insoluble support material that will lead to the reduction or total loss of mobility of the immobilized enzyme. It has many advantages, including stability, reusability, the ability to recover enzymes, and enhanced tolerance of pH and temperature [59,60,61]. Gholami-Borujeni et al. reported that immobilized horseradish peroxidases (HRPs) on calcium alginate cell beads were more stable in regards to pH and temperature than free HRPs when degrading acid orange 7 dye [62]. On the other hand, enzyme immobilization can cause issues, such as a decrease in enzymatic performance, because of confirmational changes in the enzyme, steric hindrance, and mass transfer limitation [63,64,65]. For these reasons and more, there is a need to develop new methods and new materials that overcome the shortcomings of different immobilization methods. There are two primary immobilization techniques. One technique is to physically attach the enzyme to the support materials, and the second technique is chemical binding. Under these two techniques, there are four different methods: entrapment, adsorption, covalent attachment, and cross-linking. Figure 2 shows these different methods for enzyme immobilization.

Adsorption and entrapment are physical immobilization methods. These two methods depend on weak interactions, such as van der Waals forces and ionic binding between the support and the enzyme. Physical immobilization can help preserve enzyme activity since the native enzyme structure is not affected [66]. In adsorption, the enzyme is physically bonded to the support surface via hydrogen bonds, van der Waals forces and ionic binding. This method is reversible and is the simplest one used for immobilization. It is also affordable, easy to prepare, and the most commonly used in industrial applications for scaling-up [67]. Lee et al. demonstrated the immobilization of lipase enzyme on sol–gel dried silica using carbon nanotubes to protect the enzyme. The results revealed that immobilized lipase had better activity and a longer lifetime [68]. The adsorption method can use inorganic and organic support materials. Inorganic support materials include silica. The type of silica used on a large scale is mesoporous silica SBA-15 with hexagonal arrays of pores and a pore size from 5 to 30 nanometers in diameter [69,70]. Silica gels, which possess good thermal stability and mechanical strength, are also used as carriers. The particle size in silica gel is usually 70 to 150 μm [71]. For organic carriers, chitosan, calcium alginate, cellulose, and agarose gel are used [72,73]. For the adsorption of the enzyme on the support material to be completed successfully, specific functional groups should exist on both the enzyme and the carrier. If these groups do not exist, chemical modification can be performed to ensure successful immobilization. Chemical modification is performed using a modifying agent with at least two reactive groups. One of these groups is needed to attach the modifying group chemically to the support, and the other group is to physically interact with the enzyme for immobilization. One of the most commonly used modifying agents is glutaraldehyde, which has two aldehyde groups [74,75,76]. Another physical enzyme immobilization technique is entrapment. This method, although physical, is not reversible. The enzyme is confined in a porous matrix support, thus allowing broad products to pass through while the enzyme cannot. Types of entrapment are metal-organic frameworks (MOFs), gel/fiber entrapment and microencapsulation. Entrapment leads to a significant increase in both thermal and storage stability due to the shielding and protection of the enzyme from denaturation under harsh conditions [60,67]. Cui et al. showed that CPO immobilized on Fe_3_O_3_ magnetic nanoparticles using the entrapment method was successful in degrading more than 90% of aniline blue dye [77].

Covalent attachment and cross-linking are chemical immobilization techniques. These techniques use a covalent bond to attach the enzyme and the support material using agents such as glutaraldehyde. This can lead to conformational change in the enzyme and blocking of the active site, thus affecting the activity of the enzyme. However, this is compensated for by strong chemical bonds and rigidity. The covalent attachment method can be defined as the formation of a covalent bond between the functional groups of the enzyme (including amino, carboxylic, and hydroxyl groups) and the support material used [78]. Bilal et al. [79] used covalent attachment to immobilize HRP on a calcium alginate support. The results showed that immobilized HRP had greater enzymatic efficiency and stability than free HRP. One of the reasons that this technique leads other immobilization techniques is that covalent attachment can stop enzyme leaching, thus preserving the enzyme. Another chemical immobilization method is cross-linking, which does not use support materials or matrix; instead, it develops intermolecular cross-linkage between the enzymes used by agents such as glutaraldehyde and diazonium salt [78]. Sun et al. immobilized HRP on nanocomposites by cross-linking using diethylene glycol diglycidyl ether. Immobilized HRP showed improved durability, increased activity, reusability, and it withstood microbial attack [80]. This technique has drawbacks that include enzyme denaturation in the immobilization process, which causes a loss of catalytic activity of the enzyme. Additionally, this technique has high operational costs and presents difficulties in controlling the reaction [78].

## 4. Major Challenges and Recent Progress with Enzyme-Based Approaches

### 4.1. Stability

It is well recognized that the main challenge in using free enzymes at both laboratory and industrial scales is their low stability during storage or under harsh conditions, which include high and low pH, high temperature, organic solvents, ionic liquids and oxidizing agents such as H_2_O_2_ [81,82]. Nevertheless, enzyme immobilization as well as enzyme engineering and evolution are becoming powerful tools for the improvement of enzyme stability in different environments [83,84,85].

#### 4.1.1. Stability of Immobilized Enzymes

Various research groups have been attracted to immobilization processes as a means to overcome the low stability of free enzymes [54,57]. Regarding pH, in most cases, immobilization has resulted in the unaltered activity of the immobilized enzyme at different pH values, a shift in the optimum pH, or a broader profile with little enhancement [86,87,88,89]. Nevertheless, stability in other conditions could be greatly improved. Rong et al. synthesized a multiarmed magnetic graphene oxide (GO) composite (GO@Fe_3_O_4_@6arm-PEG-NH_2_) as a carrier to immobilize HRP. The study investigated the storage stability of immobilized HRP. During cold storage at 4 °C, the immobilized HRP preserved 85.5% of its initial activity after 30 days and 72.5% after 60 days. On the other hand, free HRP sustained 42.3% of its activity after 30 days, and it was almost inactive (10.2%) after 60 days. Moreover, immobilization resulted in a substantial improvement in the thermostability of the enzyme [86]. According to a recent study, α-amylase was successfully immobilized into a covalent organic framework (COF) synthesized through a Schiff base reaction between melamine (MM) as an amine and triformyl phloroglucinol (TP) as an aldehyde in dimethyl sulfoxide (DMSO). A simple adsorption method was used to immobilize the enzyme, resulting in better thermal stability at an elevated temperature of 90 °C compared to the free form of the enzyme [88]. Wu et al. immobilized laccase enzyme on an amino-functionalized magnetic metal-organic framework (MOF) (Fe_3_O_4_-NH_2_@MIL-101(Cr)). Laccase was immobilized by combining adsorption and covalent bonding methods. This resulted in high activity recovery for laccase, enhanced tolerance toward acidic pH, elevated temperature, and excellent thermostability and storage stability. After being stored for 28 days, the laccase enzyme preserved 98% of its initial activity. It retained 49.1% of its original activity when kept for six hours at 85 °C. In addition, the stability of the enzyme in different organic solvents (methanol, ethanol, acetonitrile, acetone, and dimethyl sulfoxide) was greatly improved. As an illustration, in 12 h, free laccase lost more than 70% of its activity in such organic solvents. Upon immobilization, approximately 80% relative activity remained when immobilized laccase was stored in methanol, ethanol, and acetonitrile. Impressively, it retained more than 90% of its activity when incubated in acetone and dimethyl sulfoxide. The laccase-MOF was used in removal of the pollutant 2,4-dichlorophenol from water. It exhibited 87% efficiency in pollutant removal from water after 12 h, with the MOF contributing to an enhanced rate of pollutant removal by adsorption in the first hour. After completion of the reaction, the immobilized enzyme could be easily removed from the reaction solution using a magnet. Additionally, the ability of the laccase-MOF to retain adsorption and degradation abilities in the presence of different salt concentrations (0.2–10.0 mg/mL) was investigated. Increasing the salt concentration affected the adsorption of the MOF but had little influence on the degradation ability of laccase, as its structural stability was enhanced upon immobilization [89].

Recently, ionic liquids (ILs) have emerged as a viable alternative to toxic, hazardous, highly flammable, and volatile organic solvents, particularly for enzyme-based applications, as ILs offer attractive advantages, including enhanced catalytic activity, solubility, and stability [90,91,92,93]. These advantages are highly dependent on the biocompatibility of ILs, as some can be toxic to the enzymes. Nevertheless, their toxicity can be minimized by altering the combinations of cations and anions and/or changing the attached substituents. In addition to the formation of microemulsions by combining enzymes with a suitable surfactant to overcome the limitations of ILs, immobilization of enzymes also has great potential to enhance the stability of enzymes in ionic liquids [82]. For example, water in an oil emulsion was used to coat laccase Y20 with poly (ethylene glycol)-block-polylactide (PEG-PLA). The activity and stability of PEG-PLA-laccase was studied in an ionic liquid, specifically, 1-ethyl-3-methylimidazolium hexafluorophosphate, and the findings showed that the enzymatic activity of free laccase was slightly higher than that of PEG-PLA-laccase. This could be attributed to the mass transfer limitation of using a polymer; however, this limitation can be overcome by using IL soluble substrates. Nevertheless, the prepared polymer-laccase exhibited much higher storage stability in ILs. For example, over 70% of the initial activity of PEG-PLA-laccase was retained after 12 h of storage in an IL at 40 °C, whereas approximately 20% of the initial activity was retained for free laccase. The authors attributed the decrease in enzymatic denaturation upon immobilization to the improvement of the glycoprotein’s structural rigidity. Although enzymatic activity was decreased, the overall productivity of immobilized laccase was enhanced in ionic liquids [94].

#### 4.1.2. Enzyme Engineering and Evolution

With the development of recombinant DNA technology and enzyme engineering, generation of customized and evolved enzymes are now possible. In enzyme engineering, key mutations are introduced that improve the enzymes’ catalytic and biophysical characteristics. Such changes might lead to enhanced stability towards extreme temperature and pH, high substrate concentrations, and tolerating different organic solvents [95]. Enzyme engineering strategies can be divided into two broad categories: directed evolution and rational design. In directed evolution, two main approaches are followed, one way by recombining related sequences randomly such as gene shuffling, the other way is by introducing random mutations in an enzyme such as error prone PCR. The advantages of such approaches are that they do not require structural information, and variations at unexpected positions can be introduced, which can be far from the active site. [96].With all the advantages of directed evolution, it is accompanied with drawbacks. The changes that are produced by such approach are usually small and multiple rounds of evolution are required, which will result in a significant number of variants being screened and tested. This technique appears to be time consuming and requires reliable and high-throughput assays [96]. 

Increasing numbers of enzymes structures and models, and biochemical and computational data have made it possible to shift from the random approaches to data driven approaches, referred to as rational design. Rational design approach usually involves site-directed mutagenesis that increases the probability of identifying valuable mutations. Such approach uses enzymes’ structures and molecular modelling information to identify specific amino acids to be mutated. This approach can also lead to de novo enzyme design in which novel enzymes can be designed by introducing an activity that was not observed previously in the enzyme or by recreating known functions with different folds. Another way enzymes can be modified is through redesigning the active site of the enzyme to gain promiscuous catalytic activities and to broaden the substrate range. Random combination of mutations at targeted sites may result in synergetic effect that might have been missed in single site mutagenesis [96]. 

In a 2016 study, a cold-active esterase-encoding gene (estS, 909 bp), from *Serratia sp.* in *E. coli*, was cloned and expressed. The study found that EstS was halo-tolerant when tested against an increasing concentration of NaCl (0–4 M), as 94% of its original activity was retained even at a salt concentration of 4 M. This was attributed to the higher proportion of acidic amino acids on the surface of EstS (e.g., Asp + Glu); therefore, higher negative charges facilitated the formation of a hydrate ion network to stabilize the structure of the enzyme at high salt concentrations. Since EstS possessed low thermal stability with residual activity of 41.23% after being incubated for 1 h at 50 °C, error-prone PCR was used to generate variants of the estS gene, namely, the 1-D5 mutant with three alterations in amino acids (A43 V, R116 W, D147N). 1-D5 exhibited improved thermal stability compared with its wild type since its residual activity increased by 20% (63.29%) under the same conditions [97]. 

Oxidoreductases, particularly peroxidases, are the most popular class of enzymes for remediation of contaminated water. Due to their high redox potentials, peroxidases catalyze oxidative reactions for a variety of organic and inorganic substrates. For peroxidases to be able to efficiently oxidize various substrates, they consume the reduction of H_2_O_2_ as an oxidizing agent [98]. Nevertheless, the concentration of H_2_O_2_ must be optimized since a higher concentration inactivates the enzymes by irreversible oxidation of their active sites [99]. Enzyme evolution has been used to overcome such limitations. For example, *Escherichia coli* osmotically inducible protein Y (OsmY) was used as an expression host to accelerate the direct extracellular secretion of DyP4 from *Pleurotus ostreatus* strain PC15. When the 2, 2′-Azino-Bis-3-Ethylbenzothiazoline-6-Sulfonic Acid (ABTS) assay was performed for the wild-type DyP (WT) and OSmY-DyP4 variants (3F6 and 4D4) with increasing concentrations of H_2_O_2_ (0.15–50.00 mM), while keeping the ABTS concentration constant at 7 mM, an apparent improvement was noticed in the optimal H_2_O_2_ concentrations of 3F6 and 4D4. This was confirmed by an increase in the IC_50_ value from 0.97 mM (WT) to 4.67 mM and 7.03 mM for 3F6 and 4D4, respectively, demonstrating a higher H_2_O_2_ tolerance [81]. 

According to the literature, the combination of site-directed mutation of amino acids based on B-factor estimations can be used to generate novel variants with useful and interesting properties [100,101]. For example, error-prone PCR with site-saturated mutagenesis guided by B-factor estimation resulted in significantly improved the thermal stability of GH11 xylanase. Xing et al. have reported generating an evolved xylanase (Xyn376) with a half-life of 410 min at 70 °C, which was 820-fold more stable than that of the wild-type enzyme [100]. Another approach taken by Dotsenko et al. involved site-directed mutatgenesis of amino acids on the surface of endoglucanase II from *Penicillium verruculosum* guided by protein surface topography and multiple sequence alignment. They also reported generating a variant mutant with significantly improved thermostability [102].

### 4.2. Recyclability and Reusability

#### 4.2.1. Immobilization on Membranes

Enzymes immobilized on synthetic or commercially available membrane supports have gained much attention in recent years. Some of the attractive properties of membrane support are a large surface area, which facilitates the attachment of enzymes, and pore size, good porosity, and structure. All these factors help the reaction mixture (contaminated wastewater) access the active sites of the enzymes [103,104]. Moreover, membrane shape and geometrical configuration can be adjusted to their required purpose [105]. More critically, membranes allow for the reusability of immobilized enzymes for multiple cycles. Handayani et al. immobilized lipase enzyme on a membrane to improve its reusability. They synthesized polyethersulfone (PES) and NH_2_-polyethersulfone (PES–NH_2_) for enzyme immobilization. Membranes with pore sizes ranging from 10 to 600 nm were fabricated based on polyethersulfone (PES) and NH_2_-polyethersulfone (PES–NH_2_) polymers to be used in a bioreactor to enhance the performance of the immobilized lipase enzyme. The activity of the immobilized enzyme was not significantly affected by the immobilization process. The reusability of this system was tested using a hydrolysis reaction between p-nitrophenyl acetate and methanol. The reusability test was then repeated four times. The results showed that the activity of the lipase enzyme immobilized on PES decreased when compared to the activity of the enzyme immobilized on PES–NH_2_, which remained constant. These results demonstrated that the lipase enzyme showed better reusability on the PES–NH_2_ membrane because of stronger attraction between the lipase enzyme and the support system [104].

#### 4.2.2. Immobilization on Solid Supports

**Figure 3:**
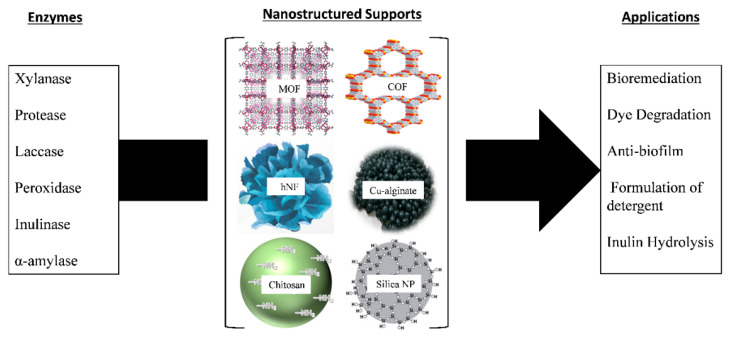
Examples of enzyme immobilization on various solid supports and their applications [95].

The criteria for solid support used for enzyme immobilization include being nontoxic, safe for the environment, inert, inexpensive, and capable of withstanding microbial attack and degradation. The solid support protects the immobilized enzyme from harsh conditions during the reaction [70,106]. All these factors, when present in solid support, enhance the reusability of the immobilized enzyme. Figure 3 shows examples of various enzymes immobilized on different solid supports and their applications.

Inorganic materials used for support for enzyme mobilization include silica and metal oxides, minerals, and carbon material. Silica is the most commonly used inorganic material for enzyme immobilization. Some of the advantages of silica include thermal and chemical resistance, high surface area, and the existence of hydroxy groups on the surface that play an important role in enzyme attachment as well as easy functionalization, making silica an ideal material for enzyme immobilization [107,108,109]. Different classes of enzymes have been immobilized on sol–gel silica, fumed silica, and silica gel [110,111]. Other types of inorganic oxides, including titanium and aluminum oxide, have been used for enzyme immobilization [112,113]. Morsi et al. [57] demonstrated that immobilized soybean peroxidase (SBP) on titanium oxide maintained 95% of its capacity to degrade the pollutant 2-mercaptobenzothiazole after four continuous reaction cycles. Siddeeg et al. [114] demonstrated that manganese peroxidase (MnP) was immobilized on Fe_3_O_4_/chitosan nanocomposites for the degradation of methylene blue (MB) and reactive orange 16 (RO 16) dyes. The reusability of these nanocomposites was tested for five consecutive cycles, where the magnetic nanocomposites were collected by a magnet and washed multiple times for the next cycle. The efficiency of removal of methylene blue dye for the five consecutive cycles was 96%, 94%, 91%, 88%, and 85%. For reactive orange 16, it was 98%, 96%, 93%, 89%, and 86%, respectively, for each of the five consecutive cycles.

New hybrid materials are another type of support material. Examples of these materials include MOFs, covalent organic frameworks (COFs), and organic-inorganic hybrid nanoflowers (hNFs). A MOF is a highly porous ordered crystalline material that is hybridized from inorganic nodes, which are metal ions or metal clusters and organic linkers that link them together [115]. These materials have been used in a variety range of applications, which include sensors, catalysis, gas separation, medical application, and water remediation [115,116,117]. Different research groups have developed several classes of metal organic frameworks which include Materials of Institute Lavoisier (MIL) [118], University of Oslo (UiO) [119], Zeolitic Imidazolate Frameworks (ZIFs) [120], and Hong Kong University of Science and Technology (HKUST) [121] with each MOF family having unique features for different applications. All these emerging materials have several excellent characteristics, such as high surface area, large pore volume, crystalline structure, and stability [122]. For these reasons and more, MOFs have been applied in environmental applications for the removal of emerging pollutants. Studies have shown that MOFs are able to act as adsorbents for different emerging pollutants. For example, ketoprofen and naproxen (both anti-inflammatory drugs) could be adsorbed on MIL-101/GO (graphene oxides) and MIL-101/GO with maximum adsorption capacity qmax of 100 and 155 mg/mL respectively [123]. Also, glyphosate, a pesticide, was effectively adsorbed on UiO-67/GO with qmax equal to 482.69 mg/g [124]. Other pollutants include naphthalene, insecticides, and tetracycline hydrochloride (TCN), an antibiotic, that have all been successfully adsorbed by different types of MOFs [125,126]. In recent years, MOFs have gained significant attention as a novel support carrier for enzyme immobilization. This is credited to the unique characteristics and features that these hybrid materials have. When it comes to enzyme immobilization on MOF, there are four immobilization approaches: physical adsorption, covalent grafting, infiltration into MOFs, and one-pot embedding. Metal organic framework immobilization using these specific techniques have both advantages and disadvantages. Some of the advantages that come with the use of a physical adsorption of enzyme to the MOF are the simplicity of the procedure. Moreover, the adsorption condition is mild and the possibility of large enzyme loading. On the other hand, some of their drawbacks are weak interaction between the enzyme and the MOF thus leading to the risk of enzyme leaching. Furthermore, they usually have low stability and reusability. For the second immobilization technique, covalent grafting, its superiority comes from the improvement of the stability and reusability of the enzyme as well as the enzyme having accessibility to the substrate. The downsides of using this technique are the complications that the synthetic reaction conditions could lead to a decrease in the enzyme activity, and high demands on time and chemicals. The third immobilization technique is infiltration into MOFs. This technique provides excellent selectivity of the substrate, better reusability, enhanced stability, decreased possibility of enzyme leaching, and superior enzyme loading. A potential drawback of this approach is that it requires strict size compatibility. The final technique is a one-pot embedding of the enzyme on the metalorganic framework. The advantages of this technique include fewer operational steps, no requirement of dimensional compatibility, enhanced reusability, decreased enzyme leaching, and better stability. Its disadvantages include partial decrease in the catalytic activity of the enzyme used, strict conditions and limited mass transfer efficiency [122,127,128]. 

**Figure 4:**
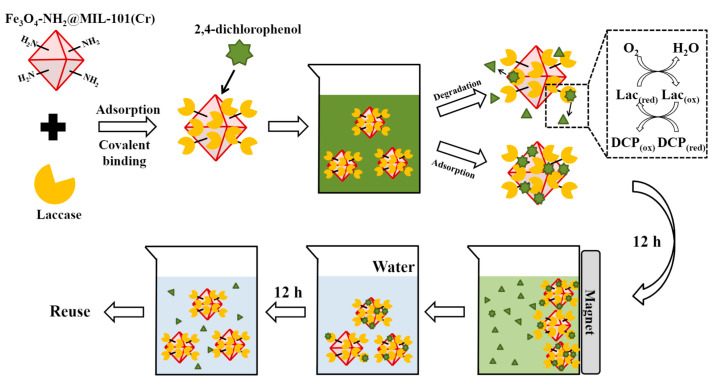
Removal of 2,4-dichlorophenol by laccase enzyme immobilization on MOF (Fe_3_O_4_-NH_2_@MIL-101(Cr)) Reprinted from [89] with permission from Elsevier. Copyright © 2021 Elsevier B.V. License Number: 5174580396809.

**Figure 5:**
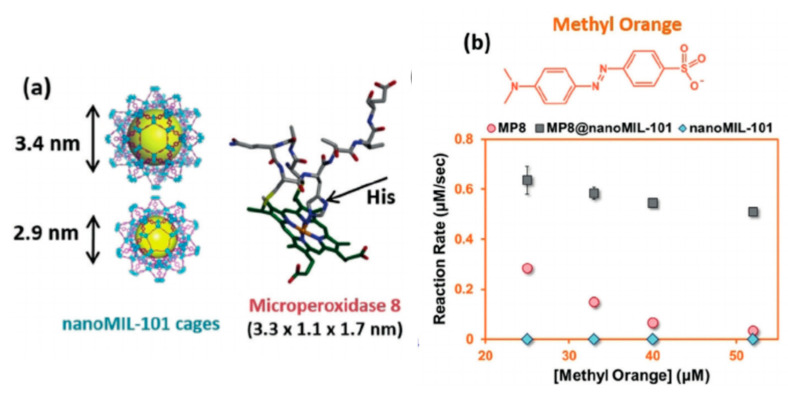
Methyl orange (MO) dye degradation of by microperoxidase (MP)-8@nanoMIL-101: (**a**) NanoMIL-101 and the structure of microperoxidase-8, and (**b**) the degradation rate of MO by MP-8@nanoMIL-101 Reprinted from [130] with permission from John Wiley and Sons. Copyright © 2021 WILEY-VCH Verlag GmbH and Co. KGaA, Weinheim. License Number: 5174580612855.

An ever-increasing body of publications have highlighted the use of enzyme-MOF composites for various water remediation applications. Gao et al. [129] synthesized MOF H-MOF(Zr) as a support material for the immobilization of HRP and CPO enzymes. The results demonstrated that hybrid composites HRP@H-MOF(Zr) and CPO@H-MOF(Zr) had 58.2% improved activity after incubation for 1 h at 70 °C compared with the free enzyme. The hybrid composite also showed great reusability, retaining 70.7% of its activity after 12 cycles. Additionally, these two enzyme-MOF hybrids demonstrated the ability to degrade 2,4-dichlorophenol and isoproturon pollutants. Wn and colleagues immobilized the laccase enzyme on an amino-functionalized magnetic MOF (Fe_3_O_4_-NH_2_@MIL-101(Cr)). The functionalized magnetic property of this MOF enhances the reusability of the enzyme. Laccase-MOF was used in the removal of 2,4-dichlorophenol pollutants from water. It exhibited 87% efficiency for pollutant removal from water after 12 h. After the completion of the reaction, the immobilized enzyme could be easily removed from the reaction solution using a magnet, making it convenient to reuse the laccase-MOF material again and again [89]. Figure 4 shows the synthesis of laccase-Fe_3_O_4_-NH_2_@MIL-101(Cr) and its reusability. Gkaniatsou et al. [130] immobilized the microperoxidase-8 (MP-8) enzyme on MIL-101(Cr) for the degradation of the harmful dye methyl orange (MO) (Figure 5). The enzyme was able to maintain 66% of its original activity after five reaction cycles. Li et al. immobilized the HRP enzyme on a highly ordered MOF (SOM-ZIF-8). The resulting hybrid HRP@SOM-ZIF-8 showed better activity, enhanced stability, and reusability. The HRP enzyme was able to sustain 85% of its initial activity after five consecutive cycles. This hybrid composite was able to degrade some known harmful dyes, methyl orange (MO), Congo red (CR), rhodamine B (RB), and rhodamine 6G (R6G), through catalytic oxidation by horseradish peroxidase immobilized on SOM-ZIF-8 [131]. Table 3 shows different examples of enzymes immobilized on MOFs for water remediation applications.

Covalent organic frameworks (COFs) are considered as the next generation of crystalline materials since they exhibit ordered structures with a tunable porosity similar to that of MOFs [132,133]. However, COFs consist of chemically/thermally stable covalent linkages and these are the only elements in their structure. Their backbones are usually made up of light elements (H, B, C, N, O, etc.), which add gravimetric advantages to these materials. They are synthesized using building blocks such as boroxine, imine, hydrazone, azine, imide, and many others. These organic building blocks can be manipulated from the atomic level where various functional groups can be added resulting in diverse designability with effective binding sites [132,134]. Therefore, COFs have been extensively used for various applications such as semiconductors, thermal insulators, luminescence and sensors, energy storage and production, adsorption and separation, as well as catalysis [134,135,136]. Recently, more interest has been given to the use of COFs in biocatalysis by incorporating catalytic enzymes into their structures. It is hypothesized that, using COFs as support materials could address the challenges presented by the use of free enzymes in real-life applications [133,137]. Samui et al., successfully synthesized TPMM COF, through a reaction between melamine (MM) as amine and tri formyl phloroglucinol (TP), successfully immobilized α-amylase onto it (via adsorption) and used it for for starch hydrolysis. TPMM-amylase showed higher thermal stability when heated up to 90 °C compared to the free form of the enzyme. It also demonstrated excellent reusability by retaining about 74% of its original activity after being reused ten times [88]. Another recent study encapsulated catalase (CAT) enzyme into COFs using an intriguing technique where digestible MOF was used to develop CAT@MOF@COF core-shell. The MOF was digested using a digesting agent, which ultimately resulted in the production of the desired COF hollow spheres (COF-42-B) containing enzyme molecules. CAT@COF-42-B could be used for the efficient conversion of hydrogen peroxide (H_2_O_2_) to O_2_ and water. The biocomposite demonstrated remarkable stability by 95% of its initial activity when exposed to acetone for 60 min compared to 25% for free CAT. Additionally, after heating at 60 °C for 10 min, free CAT and CAT@COF-42-B retained 20% and 88% of their original activities, respectively. Interstingly, CAT@ COF-42-B maintained almost all of its activity for up to 10 cycles [138].

**Figure 6:**
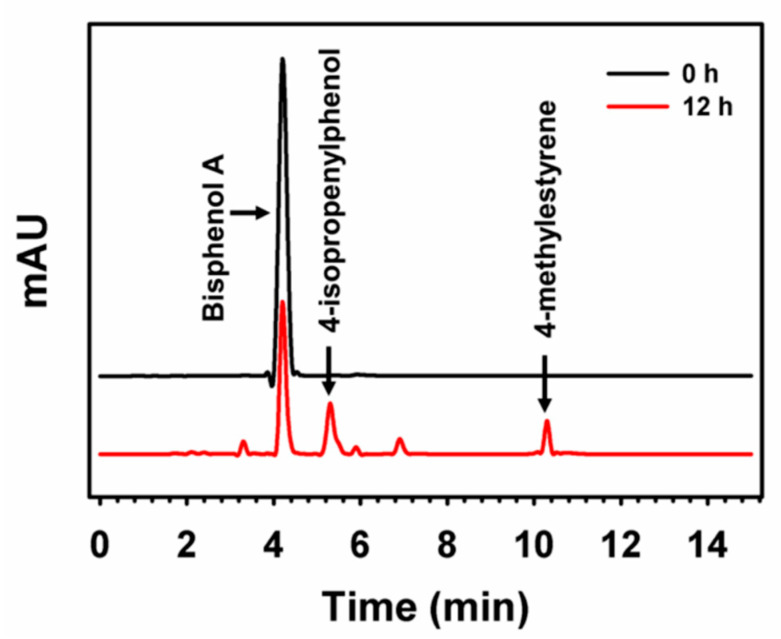
Analysis of degradation of bisphenol A by hNFs, as monitored by HPLC Reprinted from [142] with permission from the American Chemical Society. Copyright © 2021 American Chemical Society.

Another type of hybrid nanomaterial is organic–inorganic hybrid nanoflowers (hNFs). In 2012, Ge and colleagues accidentally discovered a new method for enzyme immobilization, where a flower-like structure is formed between a metal ion and an enzyme through coordination interactions [139]. One advantage of using hNFs is increased recyclability and reusability of the enzyme [140]. Fu and coworkers produced magnetic hybrid nanoflowers using laccase enzymes and copper ions. This work was achieved by binding a functionalized magnetic nanoparticle to the formed hNfs. The magnetic hNFs had a porous spherical shape, and their average diameter was 15 µm. The hNFs had great catalytic activity in the degradation of bisphenol A. They was able to degrade 100% of the pollutant in a short time of five minutes. Interestingly, the hNFs lost only 5% of their efficiency for bisphenol A degradation after five cycles [141]. Patel et al. demonstrated the formation of multimetal base hybrid nanoflowers. Novel multimetal hNFs were produced from copper and zinc ions combined with laccase enzymes. The multimetal hybrid nanoflowers had higher relative activity than zinc-laccase hNFs, copper-laccase hNFs and free laccase by 1.2-, 1.5-, and 2.6-fold, respectively. The multimetal hybrid nanoflowers were successful in degrading the pollutant bisphenol A (Figure 6). Remarkably, the remaining hybrid nanoflower activity was 1.9- and 5.1-fold higher than that for Zn-laccase hNFs and Cu-laccase hNFs, respectively, even after ten reaction cycles. [142]. Use of the CPO enzyme has been limited in industrial applications because of its poor stability and restricted reusability. Wang and coworkers synthesized hNFs using copper and cobalt ions with the CPO enzyme. They found that catalytic activity, after eight cycles, was 52.89% [143]. Rong et al. have reported producing hybrid nanoflowers from laccase enzyme and copper ions which were able to degrade more than 95% of Congo red dye [144]. Similarly, Patel et al. have syntheized a rosette shape hybrid nanoflower from laccase enzyme and copper ions [145], which were able to achieve up to 84.6% decolorization for bromophenol blue, CBBR-250 and Xylene Cyanol dyes. Altinkaynak et al. have also demonstrated preparation of hybrid nanoflowers from Turkish black radish peroxidase and copper ions which showed enhanced stability and activity in various pH values. In addition, the hybrid material was able to degrade more than 90% of Victoria Blue (VB) dye within only 1 h. Also, after the 10th cycle, the hybrid nanoflowers were still able to degrade 77% of the harmful dye [146]. Table 4 lists studies that have used hybrid nanoflowers for water remediation. All previous studies show great potential for immobilizing enzymes for use in water remediation applications, as well as the possibility of using them to scale-up in industry and wastewater treatment plants.

### 4.3. Cost

Enzymes are valuable industrial biocatalysts that have been applied in a wide range of processing and manufacturing industries [147]. The global market for industrial enzymes is projected to climb to USD 7 billion by 2023, from approximately USD 5.5 billion in 2018, with a compound annual growth rate of 4.9% [148]. This could be because industrial enzymes are considered a significant alternative to conventional chemical catalysts due to the attractive advantages they offer, including their accessibility from renewable resources, substrate and product stereochemistry selectivity, and fewer subsidiary reactions, and thus fewer waste byproducts. In addition, industrial enzymes show better catalytic efficiency than normally applied catalysts under mild pH and temperature conditions [147,149]. To date, there is limited economic analysis of enzyme-based biocatalysts, and thus, the cost-effectiveness of enzymes continues to be an intensively debated topic in industrial applications, particularly due to the harsh conditions that normally occur, namely, high pressures and temperatures, low and high pH, and oxidative environments. These conditions can easily inactivate enzymes; therefore, it is necessary to enhance the performance of these biocatalysts under the required operational conditions by improving their stability. This will increase the cost-effectiveness of their industrial implementation [83,147]. Improvements can be made using genetic engineering and recombinant DNA or immobilization technologies. In considering these technologies, it is essential to understand the overall cost and sustainability of each technique, which can be done through life cycle assessments (LCAs). Taking into consideration the chemical inputs, energy consumption, and harmful outputs, LCAs evaluate the net environmental impact of all the steps in the industrial process. Technoeconomic analysis can be used in combination with LCAs to assess the ability to apply biocatalytic techniques in large-scale processes [150,151,152].

It is well recognized that the traditional industrial techniques used to produce enzymes are generally too expensive to be widely adopted in commercial-scale processes. Nevertheless, recent advantages in biotechnology and microbiology have attracted various research groups toward genetic engineering and recombinant DNA technologies that facilitate the production of enzymes in large quantities, reduce costs, and provide tailored enzymes with enhanced properties [83,153]. Technoeconomic analysis for enzyme-based biocatalysts produced by recombinant microorganisms is being assessed for large-scale processes [152]. Recently, there has been interest in the application of these technologies in environmental remediation via the construction of highly efficient and stable enzymes with a greater ability to degrade pollutants [153]. This is done by isolating genes from novel strains of microorganisms, e.g., bacterial and fungal sources, and cloning these genes into a vector, such as a plasmid, to be expressed into a protein encoded with improved stability and catalytic activity in a suitable system [154,155]. As far as organisms are concerned, *Escherichia coli* is still the preferred choice, as it is characterized by fast growth, relatively high yields of protein, low cost, ease of handling, and versatility for producing demanding target proteins [155]. As an illustration, the study mentioned earlier succeeded in accelerating DyP4 evolution from *Pleurotus ostreatus* strain PC15 by developing a streamlined directed flow using *Escherichia coli* osmotically inducible protein Y as a bacterial extracellular protein secretion system (BENNY). This method allowed for the isolation of four genetically different strains of DyP4 with desirable properties, including higher protein yield and secretion. Hence, genetic engineering and DNA recombinant technology can be used to evolve and manipulate enzymes to be more active and stable but also to be purified easily and cheaply [81].

The cost-effectiveness can also be enhanced using immobilized enzyme-based biocatalysts. Although the immobilization process has its own cost, it produces more stable, recoverable and recyclable enzymes [150,156]. According to the LCA study, these features decreased environmental impacts via the control that immobilization provided over the materials used, energy required, and waste products produced [150]. As the prices of free enzymes depend on the source and model, these prices can range from USD 3/kg to USD 200–2000/kg. According to Sóti et al., enzyme immobilization can save up to 60% using recent technologies. However, large-scale integration of the technologies discussed here demands proper technical and economic perception [156].

### 4.4. Scaling-Up and Bioreactors

A bioreactor refers to a system that supports a biological process where a biochemical substance (enzymes, bacteria, etc.) are used. Different bioreactors have been employed for multiple applications, including the elimination of pollutants. When designing a bioreactor, two things must be considered. First, substantial research must be carried out on the biological system being used. Second, it is important to recognize the different parameters that need to be controlled, such as capital costs, installation and maintenance costs, stability, and scale-up [28,157].

**Figure 7:**
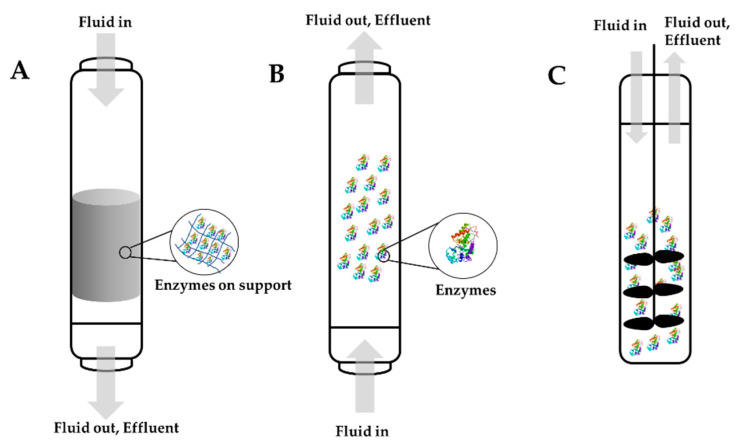
Some of the commonly used types of bioreactors: (**A**) fixed bed bioreactors, (**B**) fluidized bed bioreactors, and (**C**) stirred tank bioreactors.

The idea of enzyme immobilization reactors is based on the immobilization of the enzyme into a support ionic interaction or covalent bonds. There are several criteria to address before constructing an enzyme bioreactor. The catalytic activity of the enzyme should be at the highest possible level in regard to the units of enzyme per gram of support. Additionally, the membrane or the support used should have a second purpose. For example, it could be used to separate products and substrates. The material should be inert and have no interaction with the products and substrates, and it should provide excellent mechanical resistance. It is important to decide when the immobilized enzyme will be replaced after multiple reaction cycles. This is an important decision because it will affect the cost of production. Usually, enzymes in industrial applications will be replaced when they reach 50 to 10% of their original activity. Another essential criterion is the reaction temperature because temperature has a large impact on kinetics. Typically, immobilized enzymes have better stability over a larger range of temperatures. Last, the process needs to be simple, easy, and inexpensive [158,159]. Three main types of bioreactors can be scaled-up for enzyme immobilization on an industry-level: fixed bed reactors or packed column reactors, fluidized bed reactors, and stirred tank reactors. Figure 7 illustrates these three different bioreactors. Choosing the best option for industrial application, such as water remediation and the removal of harmful toxic emerging pollutants, will depend on the reaction kinetics and the type of support used [159].

#### 4.4.1. Fixed Bed Reactors

**Figure 8:**
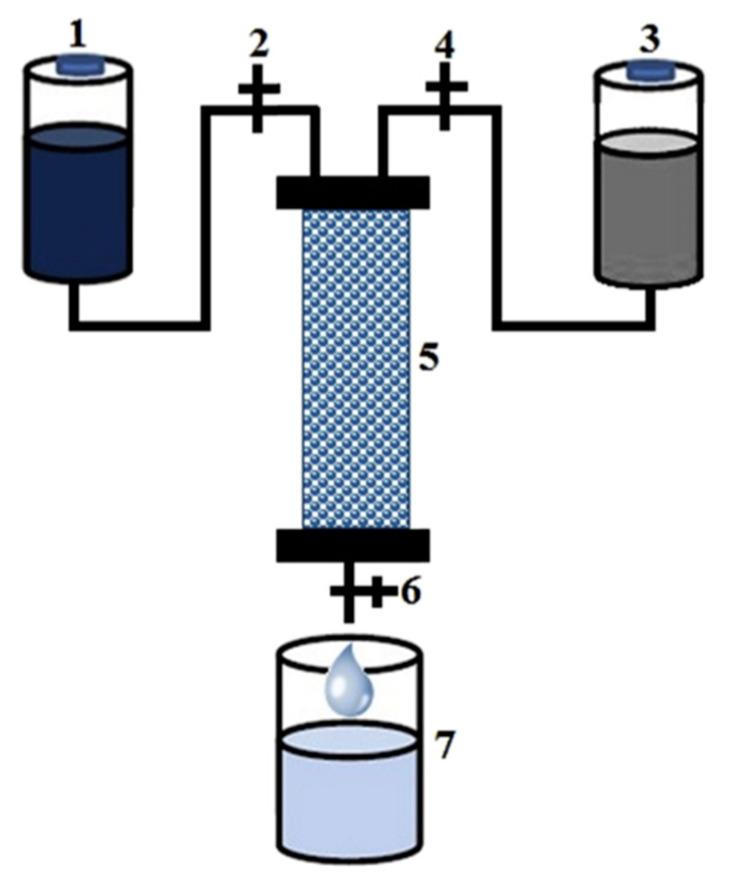
Example of a packed bed bioreactor used for dye degradation; (1) dye vessel, (2,4 and 6) flow control valves, (3) substrate vessel, (5) immobilized HRP enzyme, and (6) decolorized product Reprinted from [79] with permission from Elsevier. Copyright © 2021 Elsevier B.V. License Number: 5174590020620.

Fixed bed reactors are the most commonly used type of reactor to produce large-scale substances and intermediates. In recent years, this type of bioreactor has been used in the treatment of dangerous toxic chemicals. In fixed bed reactors, a cylindrical tube where the reaction occurs on the surface of the catalyst is placed in a fixed position (bed) in the reactor. The flow in these reactors is typically downward, where the reactants flow through the catalyst fixed bed and then transform into products [160,161,162]. There are a few examples of fixed bed reactors used in water remediation. Palmieri et al. prepared a crude laccase mixture that was immobilized by entrapping it in copper alginate beads and used for the decolorization of Remazol brilliant blue R (RBBR). The system reached 70% decolorization of this harmful dye even after 20 cycles. Different types of fixed bed reactors were tested for RBBR decolorization. The best results were obtained when the amount of laccase being loaded was reduced, and the enzyme retention was improved with the use of alginate beads coated with chitosan. The fixed bed bioreactor was constructed as follow. A glass column with a working volume of 25 mL and dimensions of 130 mm (length) × 17 mm (diameter) was packed with copper alginate beads. The solution had a flow rate of 0.2 mL/min and contained 50 µM of the dye in pH 4.5 sodium acetate buffer. The reactor system operated at approximately 20 °C [163]. Bilal et al. [79] covalently immobilized HRP on calcium alginate using glutaraldehyde for the degradation of dyes. The process was carried out in a fixed bed reactor with the immobilized enzyme and its support material, as shown in Figure 8. In brief, five grams of the biocatalyst (HRP immobilized on calcium alginate) was packed into a glass column, and the dye solution was placed into a separate vessel (Figure 1). The dye solution passed through the packed column using a pump. The solution flow rate was 2.0 mL/min. The products from the degradation by the biocatalyst were collected at the end of the outlet stream. The products were filtered and centrifuged for 15 min at 5000× *g*. The supernatant was then analyzed using spectrophotometry. For each cycle, the packed column was washed for 30 min with pH 7.0 phosphate buffer. Bilal et al. [164] used agarose beads to immobilize MnP and tested its ability for industrial application in the textile industry using a packed bed reactor system. After six sequential cycles, the analyzed effluents had a 98.4% maximum decolorization rate.

#### 4.4.2. Fluidized-Bed Reactors

**Figure 9:**
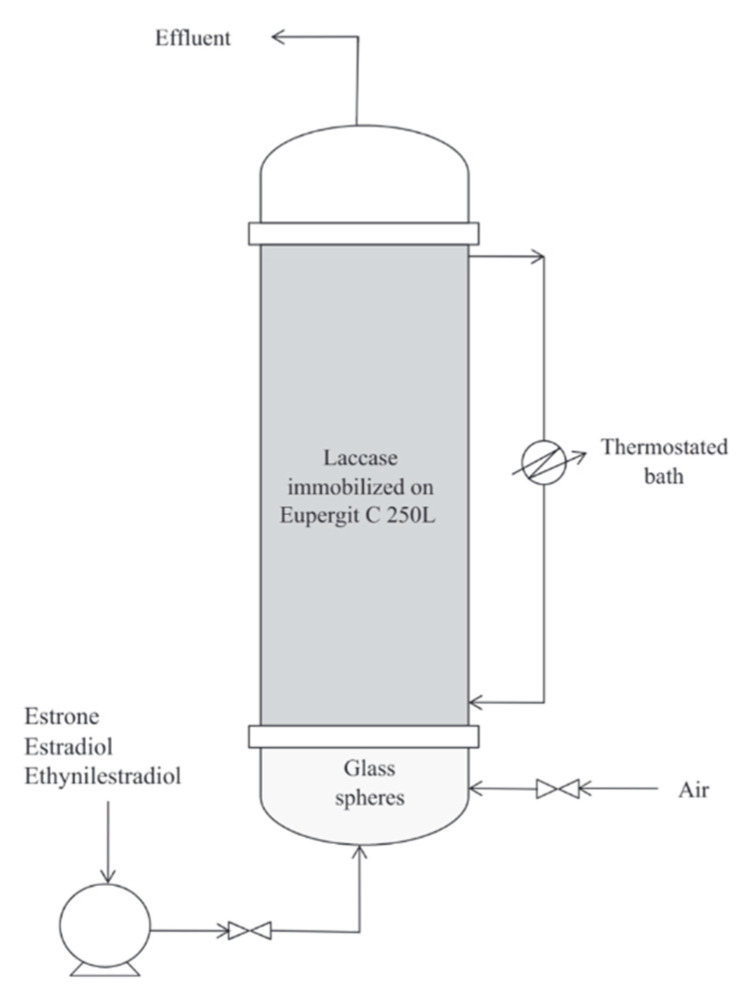
Diagram of fluidized-bed reactor with laccase enzyme immobilized on Eupergit C Reprinted from [168] with permission from Elsevier. Copyright © 2021 Elsevier B.V. License Number: 5174590199379.

Fluidized-bed reactors (FBRs) are commonly used when a reaction involves solid reactants. In this type of reactor, a fluid medium gas or liquid is passed through a solid material at a high enough speed to suspend the solid and make it act as a fluid. The advantages of using a fluidized bed reactor include high heating rates and the ability to control reaction parameters [165,166]. Piao et al. developed a fluidized bed reactor using laccase enzyme stabilized in mesoporous silica for the efficient biotransformation of bisphenol A (BPA). Glutar was used as a cross-linking agent between the laccase enzyme and porous silica. When compared with the free enzyme, the immobilized enzyme had enhanced stability over a range of temperatures and pH values. The immobilized enzyme showed high biotransformation of bisphenol A in the batch reaction. Additionally, the enzyme showed enhanced reusability without decreasing the biotransformation rate of the pollutant. The fluidized bed reactor was made of polyethylene methacrylate pipe of 10 cm in length with a 2 cm inner diameter, and was filled with the laccase enzyme immobilized on mesoporous silica [167]. In another study, Lloret et al. developed a fluidized bed reactor with immobilized laccase on a Eupergit carrier for the degradation of different types of estrogens, including estrone (E1), estradiol (E2) and ethinylestradiol (EE2). The results demonstrated high removal rates of approximately 76% and 90% for the pollutants. Moreover, the biocatalyst showed long-lasting stability over 16 days. Additionally, the activity of the estrogenic effluent was reduced up to 90%. Figure 9 shows a schematic diagram for the constructed fluidized bed reactor. The reactor was filled with 4.0 g of the enzyme with 25 U/g activity. The solution consisting of E1, E2 and EE2 in pH 7 phosphate buffer was supplied from the bottom of the bioreactor. The airflow was 0.25 L/min and the temperature was kept at 26 °C. Samples were regularly withdrawn from the bioreactor effluent for analysis of estrogen concentrations. Furthermore, 30 mg samples were taken directly from the fluidized bed reactor to determine enzyme activity. After analysis, the samples were washed and returned to the bioreactor [168].

#### 4.4.3. Stirred Tank Bioreactors

The last type of bioreactor is a stirred tank bioreactor. This reactor usually has one or more impellers attached to a shaft. Multiple parameters affect the performance of this type of reactor, such as the impeller shape, type, size, and location [169]. Lopez et al. and Flock et al. both used stirred tank bioreactors with manganese peroxidase and soybean peroxidase, respectively, for water remediation [170,171].

## 5. Conclusions

Although promising, enzyme-based remediation approaches have many major challenges that still need to be addressed. Recent progress focusing on oxidoreductases to make enzymatic wastewater treatment processes and their applications were discussed and summarized. Efficient immobilization of enzymes can circumvent some of these challenges by enhancing the stability of many enzymes and increasing the operational pH and temperature ranges. Nevertheless, there remains a need for additional research to develop new and/or hybrid materials that can address some of the drawbacks associated with currently available supports. In addition, there is still a large gap between lab level work, field research, and the scaling-up/bioreactor application for these enzymes. Future research should focus more on the real-life application of using enzymes in existing wastewater treatment plants.

## Figures and Tables

**Table 1:** Examples of some emerging pollutant (EP) classes and their descriptions.

Emerging Pollutant	Description	Examples	Reference
Pesticides	Chemical compounds that are used to manage and control pests, diseases and weeds spread. They include herbicide, insecticides, etc. They can reach the ground and surface water by irrigation.	Herbicides: 1. Aminopyralid 1. Atrazine 2. Clopyralid 3. MCPA Insecticides: 4. Chlorpyrifos 5. Heptachlor 6. Hexachlorobenzene 7. Diazinon	[13]
Pharmaceuticals	Chemical compound that are used for the treatment and/or the prevention of disease. They include anti-inflammatory drugs, analgesics and Antibiotics. Studies show not complete removal with water treatment.	Ibuprofen Sulfamethoxazole Ketoprofen Atenolol	[14,15]
Personal care products	Substance that have a widespread use and being consumed daily. They include beauty products, heath products and cleaning supplies such as shampoos, mouthwash, perfume, makeup, detergents, etc. These pollutants enter water bodies though the effluents of the sewage treatment.	Nonylphenol Salicylic acid Bisphenol A Triclosan	[16]
Food Additives	Synthesised substances such as antioxidants, thickeners, Sweeteners, Preservatives, etc. They can be found in both ground water and wastewater.	Butylhydroxytoluene (BHT) Acesulfame	[17]

**Table 2:** Different classes and concentrations of EPs detected in different water bodies.

Class	Pollutant	Max Concentration (ng/L)	Reference
Pharmaceuticals	Acetylsalicylic acid	54	[18]
	Carbamazepine	245	[18]
	Clofibric acid	68	[18]
	Diclofenac	316	[18]
	Florfenicol	111	[18]
	Flunixin	145	[18]
	Ibuprofen	376	[18]
	Ketoprofen	250	[18]
	Mefenamic acid	78	[18]
	Naproxen	321	[18]
	Metoprolol	60	[11]
	Bezafibrate	160	[11]
	Sulfasalazine	780	[11]
Hormones	Estrone	120	[18]
	17β-Estradiol	101	[18]
	17α-Ethinylestradiol	97	[18]
Personal care product	Triclosan	102	[18]
	Tonalide	66,000	[11]
	Nonylphenol	200	[11]
Pesticides	Alphamethrin	161	[12]
	Fluometuron	88	[12]
	Lambda–cyhalothrin	41	[12]
	Lindane	30	[12]

**Table 3:** Examples of different enzymes immobilized on MOF for water remediation applications.

Enzyme	Class of the Enzyme	Metal Organic Framework (MOF)	Applications	Reference
Horseradish peroxidases (HRP)	Peroxidases	H-MOF(Zr)	Degradation of 2,4-dichlorophenol pollutant	[129]
Chloroperoxidase (CPO)	Peroxidases	H-MOF(Zr)	Degradation of isoproturon pollutant	[129]
Laccase	Laccase	Fe_3_O_4_-NH_2_@MIL-101(Cr)	2,4-dichlorophenol pollutant removal	[89]
Microperoxidase-8	Peroxidases	MIL-101(Cr)	Degradation of methyl orange dye	[130]
Horseradish peroxidases (HRP)	Peroxidases	SOM-ZIF-8	Degradation of the hazardous dyes methyl orange (MO), Congo red (CR), rhodamine B (RB), and rhodamine 6G (R6G)	[131]

**Table 4:** Examples of different enzymes immobilized on hNFs for water remediation.

Enzyme	Class of Enzyme	Metal Ion	Application	Reference
Turkish black radish	peroxidases	Copper (II) ions	Dye decolorization	[146]
Chloroperoxidase (CPO)	peroxidases	Copper (II) ions	Dye decolorization	[143]
Chloroperoxidase (CPO)	peroxidases	Cobalt (II) ions	Dye decolorization	[143]
Laccase	Laccase	Copper (II) ions	Degradation of bisphenol A (BPA) pollutant	[141]
Laccase	Laccase	Copper (II) ions	Decolorization of Congo Red dye	[144]
Laccase	Laccase	Copper (II) ions	Dye decolorization	[145]
Laccase	Laccase	Multi-metal Copper (II) ions + Zinc (II) ions	Degradation of bisphenol A (BPA) pollutant	[142]

## Data Availability

Not applicable.
